# 1-Tetra­decyl­pyridinium bromide monohydrate

**DOI:** 10.1107/S1600536808039020

**Published:** 2008-11-26

**Authors:** Deborah K. Jordan, Jonathan A. Kitchen, Dylan Y. Hegh, Sally Brooker, Eng Wui Tan

**Affiliations:** a Department of Chemistry, University of Otago, PO Box 56, Dunedin, New Zealand

## Abstract

In the title compound, C_19_H_34_N^+^·Br^−^·H_2_O, the dihedral angle between the *trans*-planar alkyl side chain and the pyridinium ring is 52.73 (7)°. In the crystal structure, O—H⋯Br, C—H⋯Br and C—H⋯O hydrogen bonds form a network, while the hydro­phobic alkyl chains inter­digitate, forming bilayers.

## Related literature

For a related structure see: Vongbupnimit *et al.* (1995 ▶). For details of critical micelle concentrations in quaternary nitro­gen species, see: González-Pérez *et. al.* (2006 ▶). Fo lipid bilayers, see: Israelachvili (1985 ▶). For a discussion of hydrogen bonding, see: Desiraju & Steiner (1999 ▶).
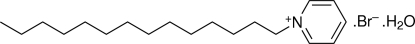


## Experimental

### 

#### Crystal data

C_19_H_34_N^+^·Br^−^·H_2_O
*M* *_r_* = 374.40
Triclinic, 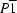

*a* = 5.5061 (13) Å
*b* = 7.4731 (18) Å
*c* = 25.039 (7) Å
α = 83.464 (15)°
β = 85.196 (14)°
γ = 79.439 (14)°
*V* = 1004.2 (4) Å^3^
*Z* = 2
Mo *K*α radiation
μ = 2.05 mm^−1^
*T* = 92 (2) K
0.21 × 0.11 × 0.02 mm

#### Data collection

Bruker Kappa APEXII CCD diffractometer
Absorption correction: multi-scan (*SADABS*; Bruker, 2006 ▶) *T*_min_ = 0.767, *T*_max_ = 0.960
16693 measured reflections
4039 independent reflections
3598 reflections with *I* > 2σ(*I*)
*R*_int_ = 0.052

#### Refinement

*R*[*F* ^2^ > 2σ(*F* ^2^)] = 0.032
*wR*(*F* ^2^) = 0.074
*S* = 1.09
4039 reflections
208 parameters
H atoms treated by a mixture of independent and constrained refinement
Δρ_max_ = 0.35 e Å^−3^
Δρ_min_ = −0.40 e Å^−3^

### 

Data collection: *APEX2* (Bruker, 2006 ▶); cell refinement: *APEX2* and *SAINT* (Bruker, 2006 ▶); data reduction: *SAINT*; program(s) used to solve structure: *SHELXS97* (Sheldrick, 2008 ▶); program(s) used to refine structure: *SHELXL97* (Sheldrick, 2008 ▶); molecular graphics: *SHELXTL* (Sheldrick, 2008 ▶); software used to prepare material for publication: *publCIF* (Westrip, 2008 ▶).

## Supplementary Material

Crystal structure: contains datablocks I, global. DOI: 10.1107/S1600536808039020/hb2826sup1.cif

Structure factors: contains datablocks I. DOI: 10.1107/S1600536808039020/hb2826Isup2.hkl

Additional supplementary materials:  crystallographic information; 3D view; checkCIF report

## Figures and Tables

**Table 1:** Hydrogen-bond geometry (Å, °)

*D*—H⋯*A*	*D*—H	H⋯*A*	*D*⋯*A*	*D*—H⋯*A*
O1—H1*X*⋯Br1	0.80 (3)	2.53 (3)	3.336 (2)	178 (3)
O1—H1*Y*⋯Br1^i^	0.77 (4)	2.56 (4)	3.330 (2)	174 (3)
C1—H1⋯Br1^ii^	0.95	2.87	3.577 (2)	133
C3—H3⋯Br1^iii^	0.95	2.85	3.783 (3)	168
C4—H4⋯O1^iii^	0.95	2.55	3.325 (3)	138
C5—H5⋯O1	0.95	2.27	3.207 (3)	171
C6—H6*B*⋯Br1^i^	0.99	2.82	3.745 (2)	155

Symmetry codes: (i) 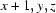; (ii) 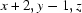; (iii) 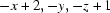.

**Fig. 1.**
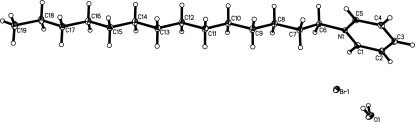
The molecular structure of (I) with 50% probability elipsoids for the non-hydrogen atoms.

**Fig. 2.**
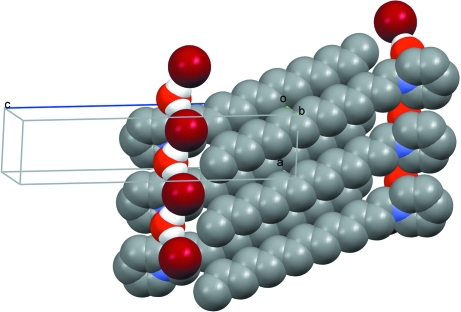
Packing of (I) viewed in the b direction. Hydrogen atoms not involved in hydrogen bonding have been removed for clarity.

## supplementary crystallographic information

### Comment

In a study to evaluate the physical properties of quaternary nitrogen species in solution, 1-tetradecylpyridinium bromide (I) was synthesized. Crystals were grown to investigate the most stable structure when the concentration of the salt was at its highest and thus suggest possible structures formed at concentrations above the critical micelle concentration, 2.77 × 10 ^-3^ mol*L*^-1^ at 298 K, (González-Pérez *et. al.*, 2006).

The asymmetric unit of (I), Figure 1, comprises the desired alkyl pyridinium cation with a bromide counter anion and a water molecule of crystallization. The hydrophobic C14 alkyl chain has a *trans*-planar arrangement. This is the expected conformation, and is very similar to that of 1-dodeylpyridinium chloride monohydrate (Vongbupnimit *et al.*, 1995). The dihedral angle formed between the alkyl chain and the pyridinium ring is 52.73 (7)°, more acute than the 79.16° seen in 1-dodecylpyridinium chloride monohydrate (Vongbupnimit *et al.*,1995). Packing in this structure, Figure 2, is governed primarily by hydrogen bonding, including both classical O—H donors and non-classical C—H donors. The bromide ion accepts a weak hydrogen bond [O1···Br1 = 3.336 (2) Å and <(O1—H1x···Br1) = 178 (3)°] from the water molecule (Desiraju & Steiner, 1999) and symmetry links it to another water molecule [O1···Br1^i^ = 3.330 (2) Å and <(O1—H1x···Br1^i^) = 174 (3)° (^i^ = *x* + 1, *y*, *z*)]. There are also five non-classical hydrogen bonds between C—H donors and either the bromide anion or the water molecule. Four of these interactions involve pyridinium C—H groups whilst one involves the methylene group attached directly to the pyridinium nitrogen, (Table 1).  Hydrophobic interactions between the alkyl chains give rise to interdigitated molecules that resemble the structure of a lipid bilayer. This may indicate that, in solution, structures at higher concentrations of the surfactant may resemble lipid bilayers and stacked lipid bilayers as suggested by Isrealachvili (1985).

### Experimental

1-Tetradecylpyridiniumbromide was synthesized by heating 1-bromotetradecane in pyridine at reflux for three hours. Excess pyridine was removed under reduced pressure and the resulting solid dissolved in a minimum of CHCl_3._ Pouring this slowly into stirring ethyl acetate resulted in the formation of a white solid which was subsequently filtered and recrystallized from methanol. The solid was dissolved in water and left to slowly evaporate, affording colourless plates of (I).

### Refinement

All H-atoms, except for water H atoms, were positioned geometrically and refined using a riding model with d(C—H) = 0.95 Å, *U*_iso_=1.2*U*_eq_ (C) for aromatic, 0.99 Å, *U*_iso_ = 1.2*U*_eq_ (C) for CH_2_ and 0.99 Å, *U*_iso_ = 1.2*U*_eq_ (C) for CH_3_ atoms. The water H-atoms were found from a difference map and refined isotropically.

### Crystal data

**Table:**

C_19_H_34_N^+^·Br^–^·H_2_O	*Z* = 2
*M**_r_* = 374.40	*F*_000_ = 400
Triclinic, *P*1	*D*_x_ = 1.238 Mg m^−^^3^
Hall symbol: -P 1	Mo *K*α radiation λ = 0.71073 Å
*a* = 5.5061 (13) Å	Cell parameters from 6262 reflections
*b* = 7.4731 (18) Å	θ = 2.5–26.1º
*c* = 25.039 (7) Å	µ = 2.05 mm^−^^1^
α = 83.464 (15)º	*T* = 92 (2) K
β = 85.196 (14)º	Plate, colourless
γ = 79.439 (14)º	0.21 × 0.11 × 0.02 mm
*V* = 1004.2 (4) Å^3^	

### Data collection

**Table:**

Bruker Kappa APEXII CCD diffractometer	4039 independent reflections
Radiation source: fine-focus sealed tube	3598 reflections with *I* > 2σ(*I*)
Monochromator: graphite	*R*_int_ = 0.052
*T* = 92(2) K	θ_max_ = 26.5º
φ and ω scans	θ_min_ = 3.1º
Absorption correction: multi-scan(SADABS; Bruker, 2006)	*h* = −6→6
*T*_min_ = 0.767, *T*_max_ = 0.960	*k* = −9→9
16693 measured reflections	*l* = −31→31

### Refinement

**Table:**

Refinement on *F*^2^	Secondary atom site location: difference Fourier map
Least-squares matrix: full	Hydrogen site location: inferred from neighbouring sites
*R*[*F*^2^ > 2σ(*F*^2^)] = 0.032	H atoms treated by a mixture of independent and constrained refinement
*wR*(*F*^2^) = 0.074	*w* = 1/[σ^2^(*F*_o_^2^) + (0.0278*P*)^2^ + 0.264*P*] where *P* = (*F*_o_^2^ + 2*F*_c_^2^)/3
*S* = 1.09	(Δ/σ)_max_ = 0.001
4039 reflections	Δρ_max_ = 0.35 e Å^−^^3^
208 parameters	Δρ_min_ = −0.40 e Å^−^^3^
Primary atom site location: structure-invariant direct methods	Extinction correction: none

### Special details

**Table:**

Geometry. All e.s.d.'s (except the e.s.d. in the dihedral angle between two l.s. planes) are estimated using the full covariance matrix. The cell e.s.d.'s are taken into account individually in the estimation of e.s.d.'s in distances, angles and torsion angles; correlations between e.s.d.'s in cell parameters are only used when they are defined by crystal symmetry. An approximate (isotropic) treatment of cell e.s.d.'s is used for estimating e.s.d.'s involving l.s. planes.
Refinement. Refinement of *F*^2^ against ALL reflections. The weighted *R*-factor *wR* and goodness of fit *S* are based on *F*^2^, conventional *R*-factors *R* are based on *F*, with *F* set to zero for negative *F*^2^. The threshold expression of *F*^2^ > σ(*F*^2^) is used only for calculating *R*-factors(gt) *etc*. and is not relevant to the choice of reflections for refinement. *R*-factors based on *F*^2^ are statistically about twice as large as those based on *F*, and *R*- factors based on ALL data will be even larger.

### Fractional atomic coordinates and isotropic or equivalent isotropic displacement parameters (Å^2^)

**Table:**

	*x*	*y*	*z*	*U*_iso_*/*U*_eq_	
N1	1.5509 (3)	−0.1133 (2)	0.38384 (7)	0.0156 (4)	
C1	1.7539 (4)	−0.2366 (3)	0.39655 (10)	0.0194 (5)	
H1	1.8848	−0.2631	0.3699	0.023*	
C2	1.7739 (4)	−0.3246 (3)	0.44777 (10)	0.0226 (5)	
H2	1.9185	−0.4104	0.4565	0.027*	
C3	1.5829 (4)	−0.2874 (3)	0.48646 (10)	0.0228 (5)	
H3	1.5947	−0.3461	0.5221	0.027*	
C4	1.3725 (4)	−0.1622 (3)	0.47226 (10)	0.0220 (5)	
H4	1.2376	−0.1362	0.4981	0.026*	
C5	1.3608 (4)	−0.0763 (3)	0.42071 (9)	0.0196 (5)	
H5	1.2177	0.0096	0.4111	0.024*	
C6	1.5377 (4)	−0.0140 (3)	0.32897 (9)	0.0192 (5)	
H6A	1.6922	−0.0566	0.3074	0.023*	
H6B	1.5281	0.1182	0.3319	0.023*	
C7	1.3178 (4)	−0.0397 (3)	0.29934 (9)	0.0198 (5)	
H7A	1.1616	0.0101	0.3193	0.024*	
H7B	1.3214	−0.1718	0.2974	0.024*	
C8	1.3274 (4)	0.0584 (3)	0.24257 (9)	0.0204 (5)	
H8A	1.3255	0.1899	0.2452	0.025*	
H8B	1.4860	0.0091	0.2235	0.025*	
C9	1.1161 (4)	0.0407 (3)	0.20918 (9)	0.0217 (5)	
H9A	1.1166	−0.0907	0.2067	0.026*	
H9B	0.9572	0.0917	0.2279	0.026*	
C10	1.1310 (4)	0.1377 (3)	0.15259 (9)	0.0206 (5)	
H10A	1.2915	0.0880	0.1343	0.025*	
H10B	1.1285	0.2692	0.1552	0.025*	
C11	0.9233 (4)	0.1197 (3)	0.11784 (9)	0.0219 (5)	
H11A	0.9264	−0.0116	0.1148	0.026*	
H11B	0.7625	0.1688	0.1362	0.026*	
C12	0.9401 (4)	0.2189 (3)	0.06138 (9)	0.0210 (5)	
H12A	1.1014	0.1703	0.0432	0.025*	
H12B	0.9363	0.3503	0.0644	0.025*	
C13	0.7339 (4)	0.2011 (3)	0.02618 (9)	0.0214 (5)	
H13A	0.7384	0.0698	0.0228	0.026*	
H13B	0.5725	0.2490	0.0444	0.026*	
C14	0.7506 (4)	0.3014 (3)	−0.02994 (9)	0.0215 (5)	
H14A	0.9122	0.2535	−0.0482	0.026*	
H14B	0.7463	0.4326	−0.0266	0.026*	
C15	0.5455 (4)	0.2843 (3)	−0.06533 (9)	0.0207 (5)	
H15A	0.5497	0.1531	−0.0687	0.025*	
H15B	0.3839	0.3323	−0.0471	0.025*	
C16	0.5619 (4)	0.3841 (3)	−0.12136 (9)	0.0211 (5)	
H16A	0.7232	0.3358	−0.1396	0.025*	
H16B	0.5584	0.5153	−0.1180	0.025*	
C17	0.3558 (4)	0.3677 (3)	−0.15676 (9)	0.0198 (5)	
H17A	0.3605	0.2366	−0.1604	0.024*	
H17B	0.1944	0.4147	−0.1383	0.024*	
C18	0.3709 (4)	0.4697 (3)	−0.21286 (9)	0.0236 (5)	
H18A	0.5315	0.4219	−0.2315	0.028*	
H18B	0.3672	0.6007	−0.2093	0.028*	
C19	0.1630 (5)	0.4532 (3)	−0.24755 (10)	0.0257 (5)	
H19A	0.1661	0.3239	−0.2517	0.031*	
H19B	0.1858	0.5201	−0.2831	0.031*	
H19C	0.0033	0.5048	−0.2302	0.031*	
O1	0.8690 (4)	0.2223 (3)	0.40217 (8)	0.0278 (4)	
H1X	0.738 (6)	0.283 (4)	0.3942 (12)	0.042 (9)*	
H1Y	0.966 (7)	0.285 (5)	0.3937 (14)	0.054 (12)*	
Br1	0.32197 (4)	0.46172 (3)	0.367608 (9)	0.02012 (8)	

### Atomic displacement parameters (Å^2^)

**Table:**

	*U*^11^	*U*^22^	*U*^33^	*U*^12^	*U*^13^	*U*^23^
N1	0.0170 (9)	0.0141 (9)	0.0155 (10)	−0.0011 (8)	−0.0041 (8)	−0.0013 (7)
C1	0.0159 (11)	0.0191 (12)	0.0223 (13)	0.0003 (9)	−0.0026 (9)	−0.0025 (9)
C2	0.0218 (12)	0.0221 (12)	0.0228 (13)	0.0012 (10)	−0.0089 (10)	−0.0009 (10)
C3	0.0312 (13)	0.0190 (12)	0.0191 (13)	−0.0060 (10)	−0.0075 (10)	0.0015 (10)
C4	0.0235 (12)	0.0225 (12)	0.0198 (13)	−0.0026 (10)	−0.0003 (10)	−0.0044 (10)
C5	0.0179 (11)	0.0191 (11)	0.0218 (13)	−0.0001 (9)	−0.0038 (9)	−0.0055 (9)
C6	0.0223 (12)	0.0183 (11)	0.0167 (12)	−0.0035 (10)	−0.0037 (9)	0.0008 (9)
C7	0.0190 (11)	0.0206 (12)	0.0199 (13)	−0.0039 (10)	−0.0049 (9)	0.0009 (9)
C8	0.0195 (12)	0.0225 (12)	0.0195 (13)	−0.0052 (10)	−0.0034 (9)	0.0010 (10)
C9	0.0219 (12)	0.0237 (12)	0.0202 (13)	−0.0079 (10)	−0.0047 (10)	0.0033 (10)
C10	0.0194 (12)	0.0222 (12)	0.0203 (13)	−0.0051 (10)	−0.0031 (10)	0.0013 (10)
C11	0.0221 (12)	0.0230 (12)	0.0211 (13)	−0.0066 (10)	−0.0052 (10)	0.0023 (10)
C12	0.0200 (12)	0.0239 (12)	0.0190 (13)	−0.0058 (10)	−0.0027 (10)	0.0026 (10)
C13	0.0211 (12)	0.0241 (12)	0.0200 (13)	−0.0071 (10)	−0.0060 (10)	0.0023 (10)
C14	0.0205 (12)	0.0256 (13)	0.0189 (13)	−0.0066 (10)	−0.0038 (10)	0.0023 (10)
C15	0.0221 (12)	0.0217 (12)	0.0193 (13)	−0.0075 (10)	−0.0040 (10)	0.0021 (10)
C16	0.0199 (12)	0.0238 (12)	0.0197 (13)	−0.0056 (10)	−0.0031 (10)	0.0016 (10)
C17	0.0203 (12)	0.0209 (12)	0.0181 (12)	−0.0046 (10)	−0.0024 (9)	0.0009 (9)
C18	0.0238 (12)	0.0310 (13)	0.0160 (12)	−0.0060 (11)	−0.0027 (10)	0.0009 (10)
C19	0.0279 (13)	0.0310 (14)	0.0187 (13)	−0.0081 (11)	−0.0040 (10)	0.0026 (10)
O1	0.0181 (9)	0.0226 (10)	0.0397 (12)	0.0006 (9)	−0.0037 (8)	0.0046 (8)
Br1	0.01596 (12)	0.02031 (13)	0.02261 (14)	0.00038 (9)	−0.00376 (9)	0.00022 (9)

### Geometric parameters (Å, °)

**Table:**

N1—C1	1.347 (3)	C11—H11A	0.9900
N1—C5	1.347 (3)	C11—H11B	0.9900
N1—C6	1.486 (3)	C12—C13	1.527 (3)
C1—C2	1.376 (3)	C12—H12A	0.9900
C1—H1	0.9500	C12—H12B	0.9900
C2—C3	1.380 (3)	C13—C14	1.519 (3)
C2—H2	0.9500	C13—H13A	0.9900
C3—C4	1.393 (3)	C13—H13B	0.9900
C3—H3	0.9500	C14—C15	1.524 (3)
C4—C5	1.376 (3)	C14—H14A	0.9900
C4—H4	0.9500	C14—H14B	0.9900
C5—H5	0.9500	C15—C16	1.516 (3)
C6—C7	1.524 (3)	C15—H15A	0.9900
C6—H6A	0.9900	C15—H15B	0.9900
C6—H6B	0.9900	C16—C17	1.528 (3)
C7—C8	1.525 (3)	C16—H16A	0.9900
C7—H7A	0.9900	C16—H16B	0.9900
C7—H7B	0.9900	C17—C18	1.523 (3)
C8—C9	1.522 (3)	C17—H17A	0.9900
C8—H8A	0.9900	C17—H17B	0.9900
C8—H8B	0.9900	C18—C19	1.524 (3)
C9—C10	1.519 (3)	C18—H18A	0.9900
C9—H9A	0.9900	C18—H18B	0.9900
C9—H9B	0.9900	C19—H19A	0.9800
C10—C11	1.527 (3)	C19—H19B	0.9800
C10—H10A	0.9900	C19—H19C	0.9800
C10—H10B	0.9900	O1—H1X	0.80 (3)
C11—C12	1.524 (3)	O1—H1Y	0.77 (4)
			
C1—N1—C5	120.7 (2)	C10—C11—H11B	108.8
C1—N1—C6	119.90 (19)	H11A—C11—H11B	107.7
C5—N1—C6	119.42 (18)	C11—C12—C13	114.38 (18)
N1—C1—C2	120.8 (2)	C11—C12—H12A	108.7
N1—C1—H1	119.6	C13—C12—H12A	108.7
C2—C1—H1	119.6	C11—C12—H12B	108.7
C1—C2—C3	119.7 (2)	C13—C12—H12B	108.7
C1—C2—H2	120.2	H12A—C12—H12B	107.6
C3—C2—H2	120.2	C14—C13—C12	114.16 (19)
C2—C3—C4	118.7 (2)	C14—C13—H13A	108.7
C2—C3—H3	120.6	C12—C13—H13A	108.7
C4—C3—H3	120.6	C14—C13—H13B	108.7
C5—C4—C3	119.8 (2)	C12—C13—H13B	108.7
C5—C4—H4	120.1	H13A—C13—H13B	107.6
C3—C4—H4	120.1	C13—C14—C15	114.50 (19)
N1—C5—C4	120.4 (2)	C13—C14—H14A	108.6
N1—C5—H5	119.8	C15—C14—H14A	108.6
C4—C5—H5	119.8	C13—C14—H14B	108.6
N1—C6—C7	113.80 (18)	C15—C14—H14B	108.6
N1—C6—H6A	108.8	H14A—C14—H14B	107.6
C7—C6—H6A	108.8	C16—C15—C14	114.58 (18)
N1—C6—H6B	108.8	C16—C15—H15A	108.6
C7—C6—H6B	108.8	C14—C15—H15A	108.6
H6A—C6—H6B	107.7	C16—C15—H15B	108.6
C6—C7—C8	110.00 (18)	C14—C15—H15B	108.6
C6—C7—H7A	109.7	H15A—C15—H15B	107.6
C8—C7—H7A	109.7	C15—C16—C17	114.58 (19)
C6—C7—H7B	109.7	C15—C16—H16A	108.6
C8—C7—H7B	109.7	C17—C16—H16A	108.6
H7A—C7—H7B	108.2	C15—C16—H16B	108.6
C9—C8—C7	114.39 (19)	C17—C16—H16B	108.6
C9—C8—H8A	108.7	H16A—C16—H16B	107.6
C7—C8—H8A	108.7	C18—C17—C16	114.54 (19)
C9—C8—H8B	108.7	C18—C17—H17A	108.6
C7—C8—H8B	108.7	C16—C17—H17A	108.6
H8A—C8—H8B	107.6	C18—C17—H17B	108.6
C10—C9—C8	113.48 (19)	C16—C17—H17B	108.6
C10—C9—H9A	108.9	H17A—C17—H17B	107.6
C8—C9—H9A	108.9	C17—C18—C19	113.9 (2)
C10—C9—H9B	108.9	C17—C18—H18A	108.8
C8—C9—H9B	108.9	C19—C18—H18A	108.8
H9A—C9—H9B	107.7	C17—C18—H18B	108.8
C9—C10—C11	114.64 (19)	C19—C18—H18B	108.8
C9—C10—H10A	108.6	H18A—C18—H18B	107.7
C11—C10—H10A	108.6	C18—C19—H19A	109.5
C9—C10—H10B	108.6	C18—C19—H19B	109.5
C11—C10—H10B	108.6	H19A—C19—H19B	109.5
H10A—C10—H10B	107.6	C18—C19—H19C	109.5
C12—C11—C10	113.93 (19)	H19A—C19—H19C	109.5
C12—C11—H11A	108.8	H19B—C19—H19C	109.5
C10—C11—H11A	108.8	H1X—O1—H1Y	105 (3)
C12—C11—H11B	108.8		
			
C5—N1—C1—C2	−1.4 (3)	C6—C7—C8—C9	179.69 (19)
C6—N1—C1—C2	177.37 (19)	C7—C8—C9—C10	−179.33 (19)
N1—C1—C2—C3	0.6 (3)	C8—C9—C10—C11	179.1 (2)
C1—C2—C3—C4	0.6 (3)	C9—C10—C11—C12	179.6 (2)
C2—C3—C4—C5	−1.1 (3)	C10—C11—C12—C13	179.72 (19)
C1—N1—C5—C4	0.9 (3)	C11—C12—C13—C14	179.6 (2)
C6—N1—C5—C4	−177.9 (2)	C12—C13—C14—C15	−180.0 (2)
C3—C4—C5—N1	0.4 (3)	C13—C14—C15—C16	−179.9 (2)
C1—N1—C6—C7	122.1 (2)	C14—C15—C16—C17	−179.78 (19)
C5—N1—C6—C7	−59.1 (3)	C15—C16—C17—C18	179.4 (2)
N1—C6—C7—C8	−177.00 (18)	C16—C17—C18—C19	−179.6 (2)

### Hydrogen-bond geometry (Å, °)

**Table:**

*D*—H···*A*	*D*—H	H···*A*	*D*···*A*	*D*—H···*A*
O1—H1X···Br1	0.80 (3)	2.53 (3)	3.336 (2)	178 (3)
O1—H1Y···Br1^i^	0.77 (4)	2.56 (4)	3.330 (2)	174 (3)
C1—H1···Br1^ii^	0.95	2.87	3.577 (2)	133
C3—H3···Br1^iii^	0.95	2.85	3.783 (3)	168
C4—H4···O1^iii^	0.95	2.55	3.325 (3)	138
C5—H5···O1	0.95	2.27	3.207 (3)	171
C6—H6B···Br1^i^	0.99	2.82	3.745 (2)	155

Symmetry codes: (i) *x*+1, *y*, *z*; (ii) *x*+2, *y*−1, *z*; (iii) −*x*+2, −*y*, −*z*+1.
